# Case Report: Combined CDK4/6 and MEK Inhibition in Refractory CDKN2A and NRAS Mutant Melanoma

**DOI:** 10.3389/fonc.2021.643156

**Published:** 2021-03-01

**Authors:** Andrea Forschner, Tobias Sinnberg, Gabi Mroz, Christopher Schroeder, Christian Philipp Reinert, Sergios Gatidis, Michael Bitzer, Thomas Eigentler, Claus Garbe, Heike Niessner, Martin Röcken, Cristiana Roggia, Sorin Armeanu-Ebinger, Olaf Riess, Sven Mattern, Dominik Nann, Irina Bonzheim

**Affiliations:** ^1^ Department of Dermatology, University Hospital Tübingen, Tübingen, Germany; ^2^ iFIT Cluster of Excellence (EXC 2180), University of Tübingen, Tübingen, Germany; ^3^ Institute of Medical Genetics and Applied Genomics, University Hospital Tübingen, Tübingen, Germany; ^4^ Department of Diagnostic and Interventional Radiology, University Hospital Tübingen, Tübingen, Germany; ^5^ Department of Internal Medicine, University Hospital Tübingen, Tübingen, Germany; ^6^ German Deutsche Forschungsgemeinschaft (DFG) Next Generation Sequencing (NGS) Competence Center, NGS Competence Center Tübingen (NCCT), Tübingen, Germany; ^7^ Institute of Pathology and Neuropathology, University Hospital Tübingen, Tübingen, Germany

**Keywords:** melanoma, *CDKN2A*, *NRAS*, ribociclib, binimetinib

## Abstract

There are only limited treatment options for metastatic *NRAS* mutant melanoma patients with resistance to immune checkpoint inhibitors. Besides activation of the mitogen-activated protein (MAP) kinase pathway, they often have additional disturbances in cell cycle regulation. However, unlike *BRAF* mutant melanoma, no targeted therapy has yet been approved for *NRAS* mutant melanoma so far. Here we present a *NRAS* mutant melanoma patient with response to combined binimetinib and ribociclib therapy following characterization of the molecular defects of the tumor by panel sequencing. Next generation sequencing (708 cancer genes) of a soft tissue metastasis revealed a homozygous deletion of *CDKN2A* in addition to the previously known *NRAS* mutation, as well as amplification of *CCNE1* and *CDK6.* Immunohistochemical staining of the altered cell cycle genes confirmed loss of p16, reduced expression of p21 and high expression of CDK6 and cyclin D1. As the patient had been progressive on combined immunotherapy, targeted therapy with combined MEK and CDK4/6 inhibition was initiated as recommended by the molecular tumor board. Response to treatment was monitored with PET/CT and liquid biopsy, serum LDH, and S100. In addition, a patient-derived xenograft (PDX) was used to prove the efficacy of the two drugs in combination. Furthermore, senescence-associated beta-galactosidase staining showed that more cells were senescent under the combination treatment of binimetinib and ribociclib. Our case demonstrates how an individualized, molecular-based therapeutic approach could be found based on next-generation sequencing results. Furthermore our report highlights the fruitful and efficient collaboration of dermatooncologists, human geneticists, molecular pathologists, biochemists, radiologists, and nuclear physicians. Further studies are urgently needed to expand the very limited therapeutic landscape of *NRAS* mutated melanoma.

## Introduction

In addition to the activation of the mitogen-activated protein (MAP) kinase pathway, *NRAS* mutant melanomas often have additional disturbances in cell cycle regulation (1). In contrast to *BRAF* mutant melanoma, no targeted therapy has yet been approved for *NRAS* mutant melanoma. However, in a phase III trial, patients treated with binimetinib, a MAP kinase inhibitor achieved improved progression-free survival (PFS) compared to dacarbazine treated patients, but no improvement of overall survival (2). Other authors suggested combining MEK inhibitors with CDK4/6 inhibitors to obtain not only apoptosis but also G1 cell cycle arrest in order to achieve synergistic effects (3, 4). Preclinical mouse models show that the combination of MEK and CDK4/6 inhibitors may not only induce senescence but also make immunological “cold” tumors amenable to PD-1 checkpoint blockade, leading to accumulation of CD8+ T cells in the tumor (5). Schuler and colleagues conducted a phase 1b/2 study in NRAS mutant melanoma patients with combined MEK inhibition (binimetinib) and CDK4/6 inhibition (ribociclib) (6). Four patients had a partial response and seven patients had a stable disease, resulting in a disease control rate of 11/16 (69%). Of note, all patients with partial response had concurrent *CDKN2A* alterations. Binimetinib and ribociclib showed no detectable negative drug interactions and no additional side effects were observed besides those known from the respective monotherapies (7, 8). Here we present a patient with partial response to combined binimetinib and ribociclib therapy after molecular defects had been characterized by panel sequencing.

## Methods

The patient provided written informed consent for the use of her clinical data for research purposes and for publication of this case report. The local independent Research Ethics Committee (IEC) approved the publication of patient data in the form of the case report. IEC-Project Number: 822/2020BO2. The Declaration of Helsinki was respected. Detailed methodology is available in **Supplementary Appendix**.

## Case Report

A 56 year old female melanoma patient had progressive disease after 1 year of adjuvant nivolumab therapy (480mg q28) following resection of transit and lymph node metastases. In 2011, the first diagnosis of an ulcerated nodular melanoma of the foot with a tumor thickness of 2.75mm was made, excision with 2cm safety distance and sentinel node had been tumor free. She had no comorbidities and was not taking any medication. There was no family history of melanoma.

As the patient developed liver, lymph node and soft tissue metastases, she was treated by four cycles of combined immunotherapy with ipilimumab 1mg/kg and nivolumab 3mg/kg every 3 weeks. However, within 3 months, there was a further progression with multiple liver metastases up to a diameter of 12cm. Therefore, treatment with chemosaturation was initiated, according to the recommendation of the interdisciplinary tumor board. Since the tumor was *BRAF* codon 600 wildtype, but *NRAS* mutated, nivolumab was continued as monotherapy despite progressive metastases in the pancreas and subcutaneous tissue of the abdomen. The next staging by PET/CT 2 months later revealed another extensive progression with newly detected bone and lung metastases. Only the liver metastases treated by chemosaturation remained stable.

**Figure 1:**
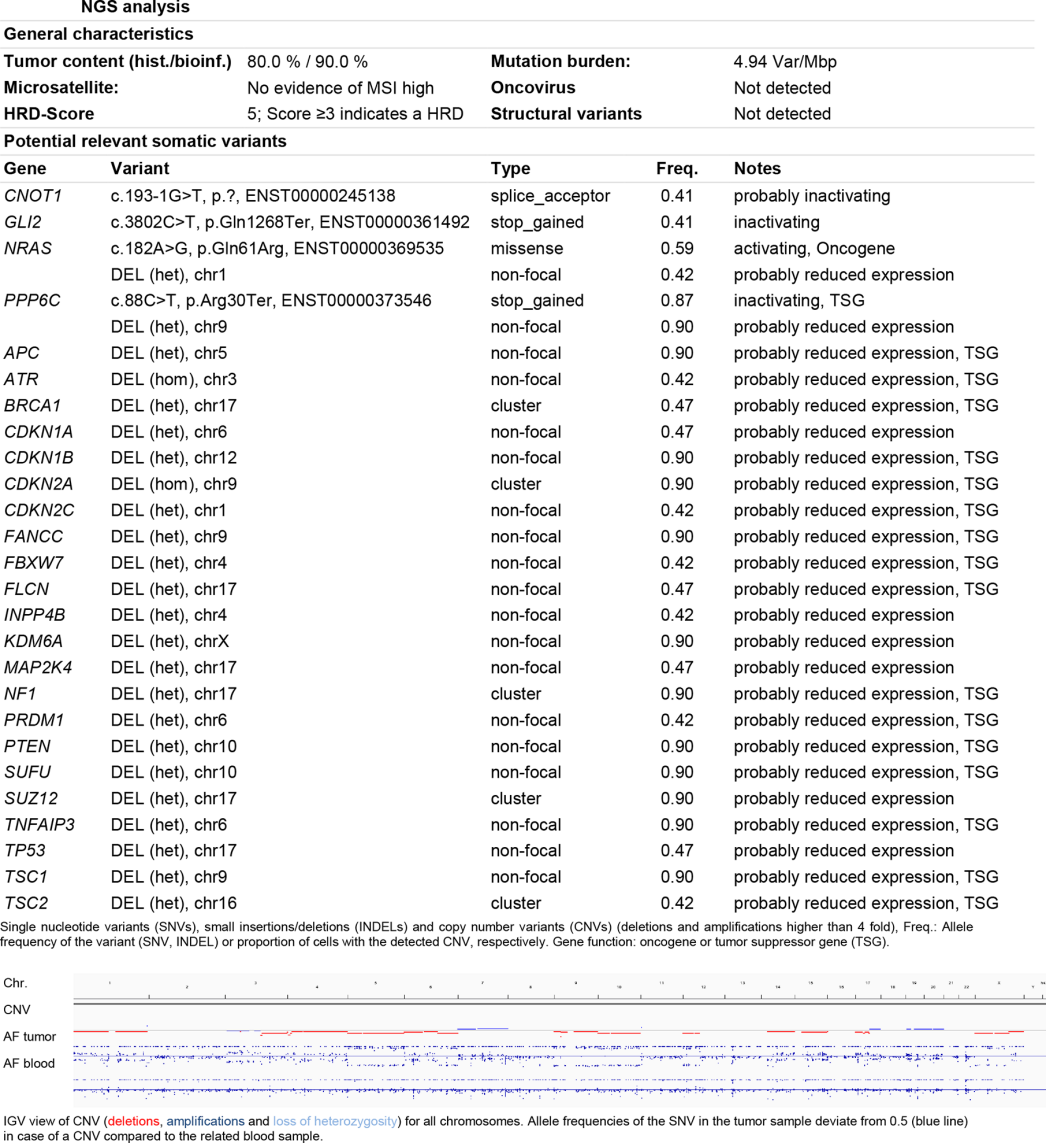
NGS analysis. General characteristics and potential relevant somatic variants were listed according to the diagnostic report. Somatic copy number variant (CNV) are shown as pictogram of the IGV view. Deletions are depicted in red, amplifications in blue. The homozygote deletion of the *CDKN2A* locus on the chromosome 9 is marked by the red dot.

Next generation sequencing (708 cancer genes) of a soft tissue metastasis revealed a homozygous deletion of *CDKN2A* in addition to the known *NRAS* mutation, and also amplification of *CCNE1* and *CDK6* (3 copies) (**Figure 1**). The tumor mutation load was 4.94 Var/Mbp.

Immunohistochemical staining of the altered cell cycle genes confirmed loss of p16, reduced expression of p21, as well as high expression of CDK6 and cyclin D1. RB1 loss was excluded, but 20% of RB were phosphorylated, confirming that the tumor had a major defect in the senescence inducing pathway (9).

According to the recommendation of the molecular tumor board, a targeted therapy with combined MEK and CDK4/6 inhibition was initiated.The treatment response was monitored with PET/CT and liquid biopsy, serum LDH, and S100. Since ribociclib 200mg per day 21d q28 in combination with binimetinib 45mg twice daily was found to be the optimal regime for melanoma therapy (6), we started this combination with exactly that dose and schedule, when echocardiography showed a normal left ventricular ejection rate.

**Figure 2:**
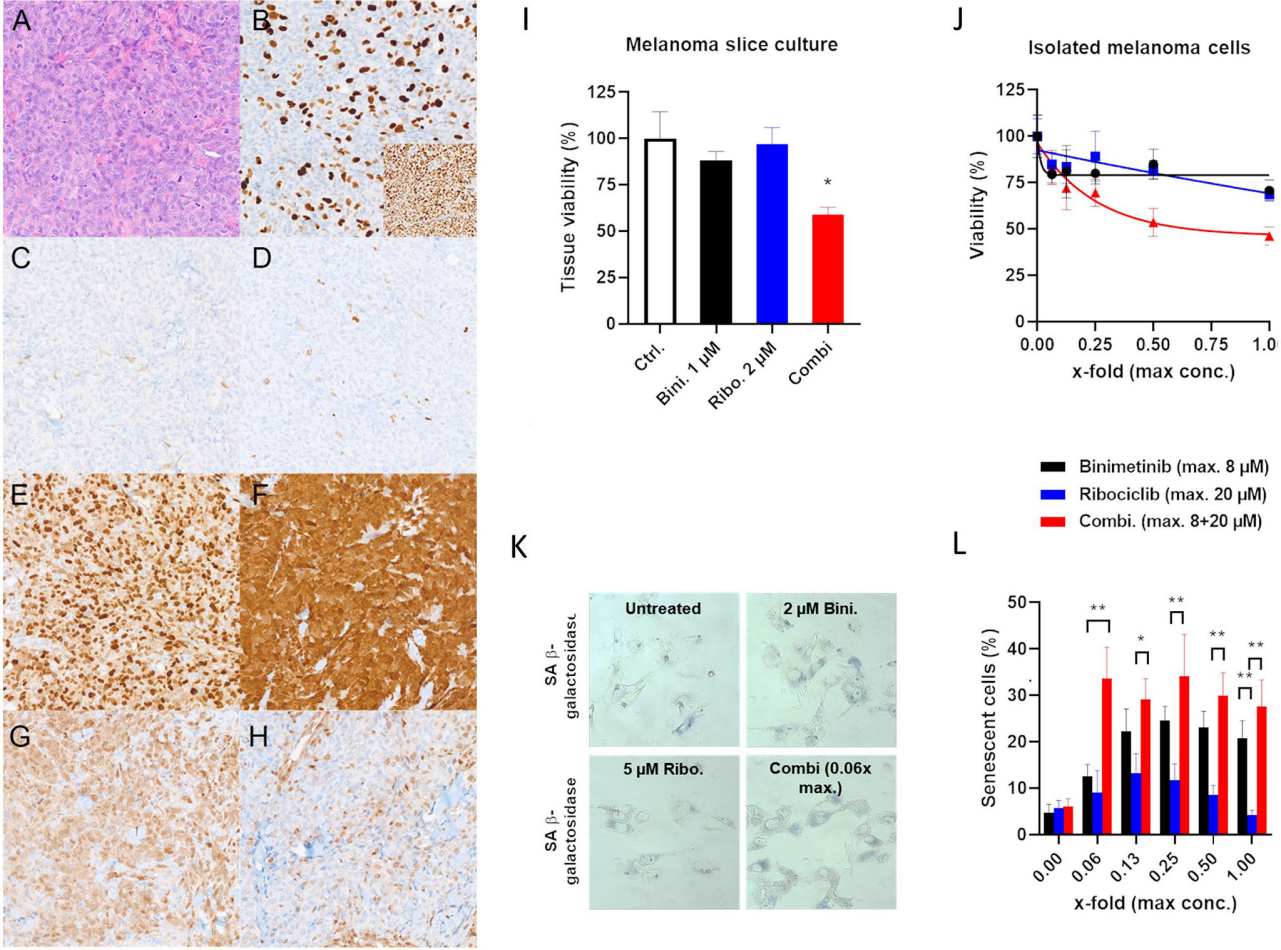
Immunohistochemical panel **(A–H). (A)** A solid tumor composed of atypical enlarged cells with increased mitotic activity, hematoxylin and eosin staining, 200x original magnification, **(B)** All cells have preserved retinoblastoma 1 protein (insert), whereas about 30% of RB is phosphorylated (main picture, phospho-RB antibody). **(C)** The tumor cells show a loss of p16 (only stroma cells are positive) and nearly all cells are negative for p21 **(D)**. **(E)** Moderate to strong positivity for cyclin D1 in the majority of the tumor cells. **(F)** Strong and homogenous positivity for CDK6 in all tumor cells. **(G)** Moderate nuclear and cytoplasmic positivity for phospho-p44/42 MAPK in a vast amount of tumor cells (primarily at the invasive front). **(H)** weak, primarily cytoplasmic positivity for phosphor-p38 MAPK in a minority of tumor cells (primarily in the invasive front). **(B–H)** Immunoperoxidase, 200x original magnification). *In vitro* models used for efficacy testing of binimetinib plus ribociclib **(I–L). (I)** Alamar blue assay of metastasis slice cultures shows reduced cell viability after combined ribociclib and binimetinib treatment: (mean +/−SD, n=4; *p < 0.05; One-way ANOVA with Sidak’s multiple comparisons test *versus* untreated controls). **(J)** Melanoma cells isolated from a patient-derived xenograft tumor generated with tumor cells of the metastasis used in subfigure I show reduced viability after combined ribociclib and binimetinib treatment (alamarBlue assay) (mean +/− SD, n=6). **(K, L)** Senescence-associated beta-galactosidase of isolated melanoma cells show more intensive staining with combined ribociclib and binimetinib treatment indicating senescence. Percentage of senescent cells is depicted as clear blue cell count normalized to the total cell count (mean +/− SD, n=6, *p < 0.05, **p < 0.01).

Immediately before starting combined binimetinib and ribociclib, a painful subcutaneous metastasis on the left axilla was surgically removed. This metastasis also underwent immunohistochemical validation of the cell cycle genes with comparable results (**Figures 2A–F**) and was used to prepare slice cultures.

Supporting the therapy with a MEK inhibitor, the immunhistochemistry for phospho-p44/42 MAPK (ERK1/2, downstream targets of MEK) showed a moderate positivity in a vast amount of tumor cells in the invasion front. Phospho-p38 MAPK showed only a weak positivity (lower than adjacent endothelial cells) (**Figures 2G, H**).

The slice cultures were treated *in vitro* with either ribociclib or binimetinib alone or with the combination. The combination reduced the viability of the tumor tissue after 4 days compared to the untreated control (**Figure 2I**).

In order to amplify ribociclib and binimetinib naïve melanoma cells, a few vital tumor cells of the subcutaneous metastasis were injected in a NOD.Cg-Prkdc^scid^ Il2rg^tm1Wjl^/SzJ (NSG) mouse. This patient-derived xenograft (PDX) was used to re-isolate melanoma cells for further *in vitro* tests after a solid tumor had grown. These cells proved the efficacy of both drugs in combination in an alamarBlue viability assay (**Figure 2J**). Furthermore, senescence-associated beta-galactosidase staining was performed after 3 days of treatment. We observed that the maximum measurable effect was about 30% of beta-galactosidase positive cells. This was found at already low concentrations by SA-b-galactosidase staining when compared to the monotherapies (**Figures 2K, L**).

Apart from an acneiform eruption that was controlled by oral tetracycline (doxycycline 100mg or later minocycline 50mg), the patient had no side effects during the first weeks of therapy.

**Figure 3:**
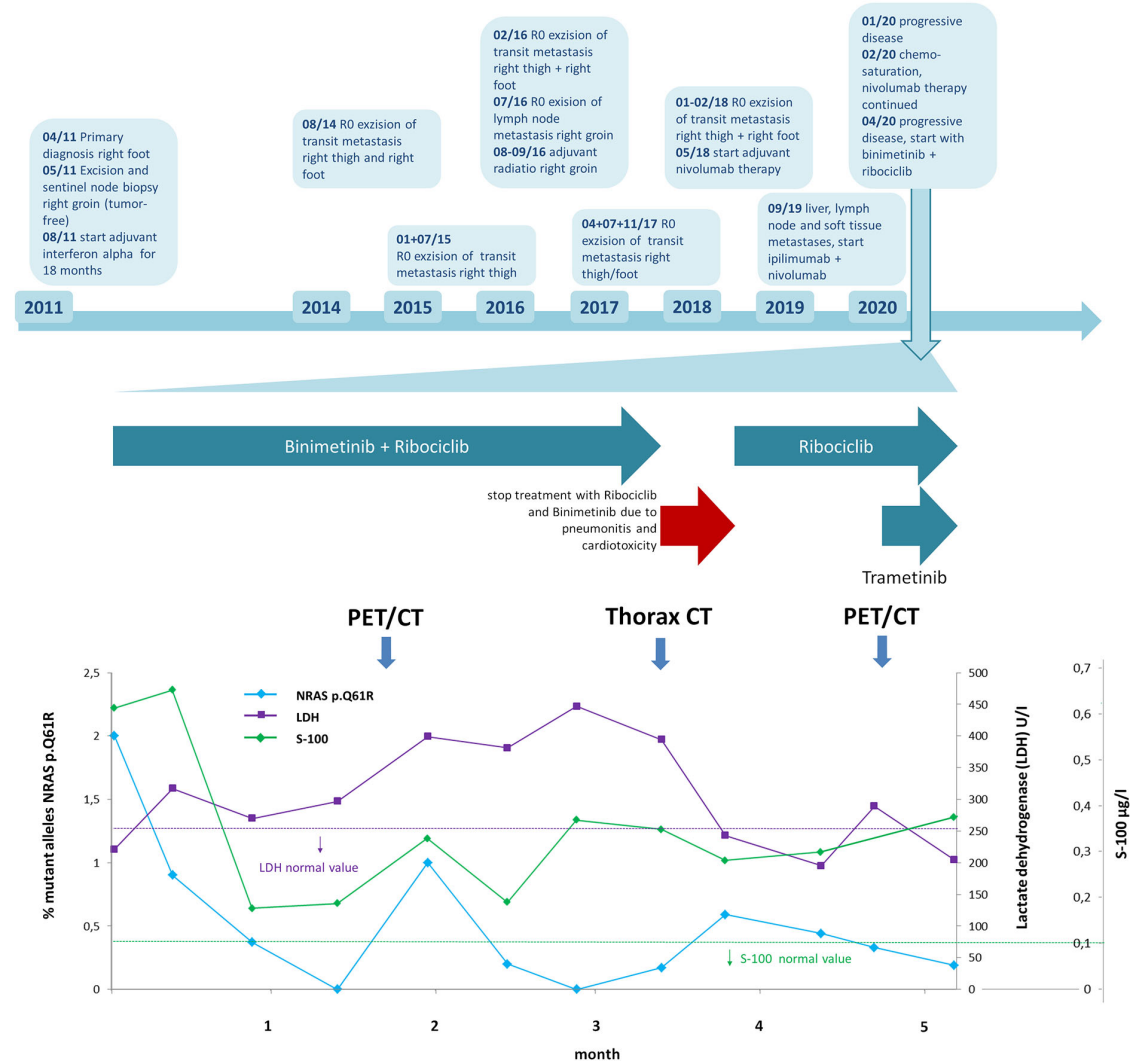
Time line of patient’s history and in detail time course of lactate dehydrogenase (LDH) and S100 levels as well as *NRAS* c.182A>G/p.Q61R allele fractions in liquid biopsy after initiation of ribociclib and binimetinib therapy. Upper part: time line depicts patient’s disease course with different treatments. Lower part (magnification of time course during therapy with ribociclib and binimetinib): monitoring shows elevated LDH and S100 levels during the course of therapy while *NRAS* c.182A>G/p.Q61R allele fraction in liquid biopsy decreased and dropped below limit of detection with deviations after six weeks and twelve weeks (analysis limit of detection 0.21–0.69%, depth 2,393–75,606, mol depth 603–3,486). LDH and S100 normal value ranges are depicted as purple and green dashed lines. Horizontal arrows indicate periods of kinase inhibitor treatment. Vertical arrows indicate time points of follow-up PET/CT and thorax CT staging.

**Figure 4:**
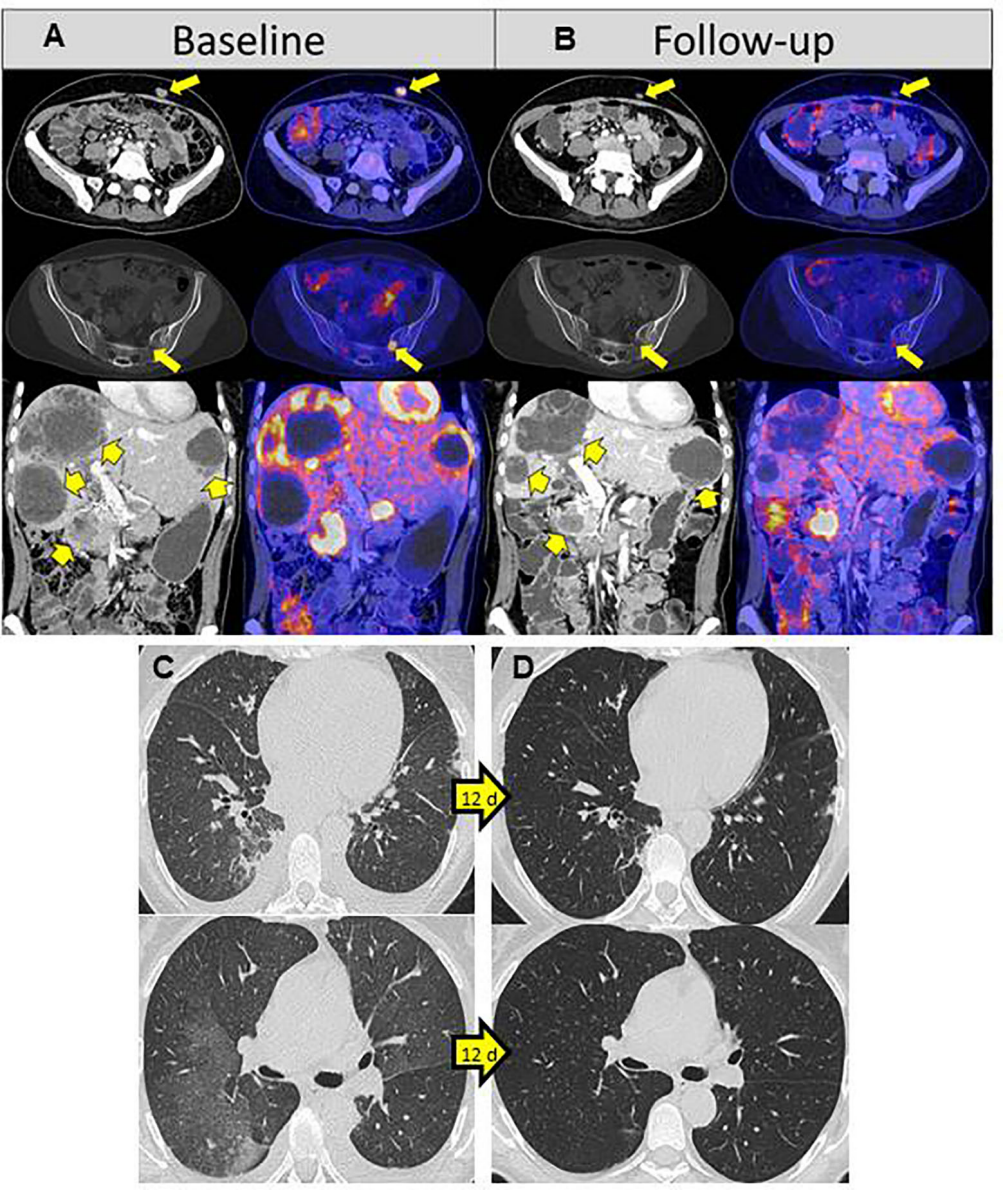
^18^F-FDG PET/CT baseline and 8 weeks of treatment with ribociclib and binimetinib **(A, B)**. **(A)** ^18^F-FDG PET/CT at baseline staging showing ^18^F-FDG avid metastases in soft tissue (upper row), sacral bone (middle row), liver, and pancreas (lower row). **(B)** At follow-up staging after 8 weeks of treatment with ribociclib und binimetinib, soft tissue metastases decreased in size and had a significantly reduced 18F-FDG uptake (upper row). The sacral bone metastasis showed an almost complete reduction of ^18^F-FDG accumulation (middle row). Pancreas and liver metastases transformed necrotic with lower amount of solid tumor masses and significantly reduced ^18^F-FDG uptake (lower row). A ^18^F-FDG avid metastasis was still observed in the pancreatic head. The whole-body tumor volumetric parameters, defined as the sum of all ^18^F-FDG avid metastases, were considerably decreased at follow-up (whole-body MTV: 20.6 cm^3^; whole-body TLG: 156.2) compared to baseline staging (whole-body MTV: 66.0 cm^3^; whole-body TLG: 582.3). Rapid recovery of pneumonitis after 12 days **(C, D)**. **(C)** Partly flat milky glass-like, partly spotty consolidating infiltrates typical findings for pneumonitis. **(D)** Complete recovery 12 days later with 100mg prednisolone.

LDH increased slightly during the course of therapy and S100 also remained significantly elevated after an initial decline. In contrast, *NRAS* mutation in the liquid biopsy, initially detectable with an allele frequency of 2%, dropped significantly to 0.9 and 0.37% after 4 weeks of therapy. Six weeks after therapy start, cell-free tumor DNA was undetectable (**Figure 3**). In line with the *NRAS* monitoring in liquid biopsy, PET/CT 8 weeks after initiation of therapy showed a remarkable reduction of whole-body vital tumor mass (**Figures 4A, B**).

However, 15 weeks after treatment initiation, the patient developed a marked deterioration in her general condition and dyspnea during minimal physical activity.

Thoracic CT revealed severe pneumonitis, probably binimetinib-induced (**Figures 4C, D**). In addition echocardiography showed a significant reduction of the left ventricular ejection fraction to 24%, most probably also caused by the current therapy. The patient was hospitalized and treated with high-dose corticosteroids: 100mg prednisolone intravenously per day. Therapy with binimetinib and ribociclib was interrupted. Furthermore, diuretics and beta-blocker were started. Just 1 day after the first steroid administration the patient felt much better and was able to climb stairs again after a few more days. Prednisolone was reduced by 20mg per week and 12 days after diagnosis, pneumonitis had completely resolved (**Figure 4D**). By this time, left ventricular ejection fraction had improved to 37%. Treatment with ribociclib was re-started in a dosage of 400mg per day 21d q28. Binimetinib was reintroduced 4 weeks later, when the echocardiography showed a normal left venticular ejection fraction of 59%. At this time, PET/CT results revealed progression on ribociclib monotherapy. We therefore re-started MEK inhibition with trametinib 0.5mg per day, one quarter of the recommended daily dose. One week later, when there were still no clinical signs of pneumonitis or cardiotoxicity, trametinib was increased to 0.75mg per day. During treatment interruption the mutant alleles of *NRAS* c.182A>G/p.Q61R had increased again to 0.59% in the cfDNA, decreasing after re-initiation of ribociclib to 0.33% and furthermore to 0.19% after supplementing trametinib (**Figure 3**).

As 14 days after the initiation of trametinib, echocardiography showed still normal left ventricular ejection fraction and the patient had no clinical signs of pneumonitis, we increased trametinib to 1mg per day under close clinical supervision. Echocardiography and thoracic CT follow-up remained stable under the treatment regime with ribociclib 400mg 21d q28 and trametinib 1mg per day without prednisolone therapy.

## Discussion

This case report demonstrates how an individual, molecular-based therapeutic approach could be found based on next-generation sequencing results. Of advantage was the already established treatment regime and dosis for the combination of binimetinib and ribociclib from a phase 1b/2 trial (6). With molecular-based off-label therapy, it is important to evaluate potential treatment success or failure of as early and as reliably as possible. We therefore performed PET/CT immediately before the start of therapy and also collected liquid biopsies in addition to the established tests such as S100 and LDH. Since fresh tissue could be obtained before starting the therapy, it was also possible to establish a PDX model to test the efficacy of the two drugs *ex vivo*. Due to the urgency with the rapid tumor growth, we performed the testing in parallel to therapy initiation. However, PDX models have their greatest importance at an earlier point in time, before the actual start of the therapy. Furthermore, the PDX model could not be used as a therapy *in vivo* model due to regulatory requirements. Together with further in-depth experiments, a deeper understanding of the interaction of binimetinib and ribociclib should be generated, which our case study cannot provide. In our patient, monotherapy with either binimetinib or ribociclib alone was less effective than the combination. This fits well with the published results: CDK4/6 inhibitors alone suppress proliferation with little effect on apoptosis, while the drug combination of MEK and CDK4/6 inhibitors induced both, apoptosis and cell cycle arrest, what should result in tumour regression (3, 4).

PET/CT is an appropriate imaging modality to assess response of such molecular-based therapeutic approaches, as it provides both, morphologic and metabolic informations of metastases (10, 11) and liquid biopsies allow specific monitoring of driver mutations during melanoma therapy (12).

The severe treatment-related adverse events of our patient, both pneumonitis and reduction of the left ventricular ejection fraction, were most likely caused by the MEK inhibitor. In the NEMO study, pneumonitis occurred in 1% of the patients treated by binimetinib and a decrease of the left ejection fraction in 4% of the patients (2). In contrast, neither a decrease in left ventricular ejection fraction nor pneumonitis was observed with CDK4/6 inhibitors (13, 14). Therefore we decided to re-start CDK4/6 inhibition earlier than MEK inhibiton. Ribociclib was re-started as soon as the pneumonitis had disappeared but as monotherapy because cardiac function had not yet fully restored. During treatment with trametinib, which was slowly increased to 50% of the recommended daily dose, there was no recurrence of cardiotoxicity or pneumonitis. This suggests that switching the drug may sometimes be helpful in managing the side effects. The progressive disease with initial ribociclib monotherapy indicates the need for combined CDK4/6 and MEK inhibition. Since further resistance mechanisms are likely to occur, we do not know how long the patient will benefit from this regimen.

This case demonstrates the fruitful and efficient collaboration of dermatooncologists, human geneticists, molecular pathologists, biochemists, radiologists and nuclear physicians. An interdisciplinary molecular tumor board is important for decision making of molecular-based off-label therapies. Registers should be established to collect decisions and outcomes of such molecular-based therapeutic strategies to facilitate the development of new treatment approaches. In addition, basket studies would be desirable to cover the costs of the therapies and to standardize monitoring. Further studies are urgently needed to expand the very limited therapeutic landscape of *NRAS* mutated melanoma.

## Data Availability Statement

The datasets presented in this study can be found in online repositories. The names of the repository/repositories and accession number(s) can be found in the article/**Supplementary Material**.

## Ethics Statement

Local Research Ethics Committee (IEC) Tuebingen approved the publication of patient data in the form of the case report. IEC-Project Number: 822/2020BO2. The patients/participants provided their written informed consent to participate in this study. Written informed consent was obtained from the individual(s) for the publication of any potentially identifiable images or data included in this article.

## Author Contributions

Drafting of the manuscript: AF, TS, and IB. Molecular and pathological analysis, tumor sequencing, liquid biopsy: CS, CR, SA-E, OR, SM, DN, and IB. *In vitro* models: TS and HN. Radiological imaging: CPR and SG. Coordination of clinical care: AF, GM, MB, TE, CG, and MR. All authors contributed to the article and approved the submitted version.

## Conflict of Interest

AF served as consultant to Roche, Novartis, MSD, BMS, Pierre-Fabre; received travel support from Roche, Novartis, BMS, Pierre-Fabre, received speaker fees from Roche, Novartis, BMS, MSD, and CeGaT outside the submitted work. She reports institutional research grants from BMS Stiftung Immunonkologie outside the submitted work. CS reports institutional grants from Novartis and grants from BMS Stiftung Immunonkologie outside the submitted work. MB served in advisory committees for Bayer, BMS, EISAI, IPSEN, and MSD outside the submitted work. TE reports personal fees from Amgen, grants and personal fees from Novartis, personal fees from Philogen, grants and personal fees from Roche, grants and personal fees from Sanofi, personal fees from BMS, personal fees from MSD, outside the submitted work. CG reports personal fees from Amgen, grants and personal fees from NeraCare, grants and personal fees from Novartis, personal fees from Philogen, grants and personal fees from Roche, grants and personal fees from Sanofi, personal fees from BMS, personal fees from MSD, outside the submitted work. IB received speaker fees from Novartis, Bayer and AstraZeneca and honoraria for advisory board participation from BMS and Novartis.

The remaining authors declare that the research was conducted in the absence of any commercial or financial relationships that could be construed as a potential conflict of interest.
