# Supplementary material for: Case Report: Combined CDK4/6 and MEK Inhibition in Refractory CDKN2A and NRAS Mutant Melanoma
Source: Front Oncol. 2021 Mar 1;11:643156. doi: 10.3389/fonc.2021.643156 (PMC7959243; doi:10.3389/fonc.2021.643156)
Supplement: Supplementary file 1 [file DataSheet_1.docx]

**Methods**

**Histopathology and IHC**

Immunohistochemistry (IHC) was performed on formalin-fixed, paraffin-embedded (FFPE) tissue sections on the Ventana Ultra XT automated staining System (Ventana Medical Systems, Oro Valley, AZ, USA) using Ventana reagents, according to the manufacturer´s protocol. Tissue sections were stained with an antibody against CDK6 (ERP4515, 1:250, Abcam, Cambridge, UK), Cyclin D1 (SP4, rtu, Roche, Mannheim, Germany), p16 (INK4a, rtu, Roche), RB1 (G3-245, 1:100, BD Pharmingen, San Diego, CA, USA), phosphor-RB (D20B12, 1:200, Cell Signaling, Danvers, MA, USA), p21 (SXM-30, 1:500,BD Pharmingen), phospho-p44/42 (ERK1/2, T202/Y204, 1:400 Cell Signaling) and phospho-p38 (T180/Y182, D3F9, 1:800, Cell Signaling). Primary antibody detection was performed using the OptiView DAB IHC detection kit (Ventana).

**Next-generation sequencing**

A total of 200 ng of extracted genomic DNA (gDNA) was used for library construction. gDNA was fragmented to 150-200 bp pairs using Covaris Ultrasonication. The SureSelect XT Low Input Target Enrichment System (Agilent Technologies, Santa Clara, CA, USA) was used to perform hybrid capture with custom-designed bait-sets Sure Select Somatic Cancer Panel v5 covering 708 cancer related genes, 7 promoter regions and selected fusions. Raw data was processed using the in-house megSAP Pipeline (<https://github.com/imgag/megSAP>, version 0.2-8-ga9d80c2) combined with ngs-bits package (https://github.com/imgag/ngs-bits). Briefly, sequencing reads were aligned to the human reference genome (GRCh37) by BWA-MEM, variants were called using Strelka2 and annotated with VEP. For SCNA detection ClinCNV (version 1.16) was used.

**Liquid Biopsy and Next Generation Sequencing for *NRAS* c.182A>G/p.Q61R monitoring**

Plasma was isolated and cfDNA was extracted from Liquid Biopsy (whole blood taken in PAXgene Blood ccfDNA System primary tubes (Qiagen, Hilden, Germany) and further processed the same day) using the QIAamp Circulating Nucleid Acid Kit (Qiagen) according to the manufacturers’ instructions.

Cell free DNA was analyzed to monitor the *NRAS* c.182A>G/p.Q61R mutation using the Oncomine Colon cfDNA Assay (Thermo Fisher Scientific, Waltham, MA, USA) and Next Generation Sequencing was performed on the Ion GeneStudio S5 prime. Library preparation and semiconductor sequencing was done according to the manufacturers’ manuals using the Ion AmpliSeq Library Kit v2.0, the Ion Library TaqMan Quantitation Kit, the Ion 510 & Ion 520 & Ion 530 Kit – Chef and the Ion 520 Chip Kit (Thermo Fisher Scientific). Variant calling of non-synonymous somatic variants compared to the human reference sequence (hg19) was performed using Ion Reporter Software (Thermo Fisher Scientific, Version 5.12.3.0). *NRAS* codon 61 mutations status was revised on raw data level in the Integrative Genomics Viewer (IGV; Broad Institute, Cam-bridge, MA; Version 2.5.2).

**Data availability**

Sequencing data are deposited in the European Nucleotide Archive (ENA) under accession number PRJEB40791 (https://www.ebi.ac.uk/ena/).

**Slice culture model and alamarBlue viability assay**

A punch biopsy of the soft tissue metastasis was cut into 400 µm thick slices using a vibratome (Leica VT1200S). The slices were cultured in cell culture medium for four days in quadruplicates and treated with either binimetinib, ribociclib or the combination. After treatment for four days, an alamarBlue Cell Viability Assay (Thermo Fisher Scientific) was performed. Therefore, the supernatant of the tissue was removed and in each new cell culture medium was added containing 0.01 mg/ml alamarBlue and incubated at 37°C for 2 h. The fluorescence intensity (λex = 540 nm and λem = 640 nm) was measured using a microplate fluorimeter (Tristar, Berthold) and normalized to untreated control slices (set to 100%).

**Tumor propagation and tumor cell isolation for 2D viability assays and senescence-associated beta-galactosidase stainings**

A piece of the soft tissue metastasis was cut into 1-2 mm fragments and digested using an enzymatic mixture as formerly described [15]. Next, a single cell suspension was prepared and subcutaneously injected into a female a NOD.Cg-Prkdcˢᶜⁱᵈ Il2rgᵗᵐ¹ᵂʲˡ / SzJ  (NSG) mouse to propagate the tumor tissue as patient-derived xenograft/ PDX (approval HT07/18A). After the tumor had grown to >12 mm in diameter, the tumor was excised and a single cell suspension prepared as described above. Thereof, a melanoma cell culture was prepared using RPMI-1640 containing 10% fetal calf serum and penicillin-streptomycin (100 units/ml). 2,500 cells per well were used to perform an alamarBlue viability assay in the 96well format and treating the cells for 72 hours. The assay was done in six replicates. Binimetinib and ribociclib (HY15202 and HY15777, all from MedChemExpress/ Hycultec) were dissolved in DMSO as 10 mM stock solutions.

Senescence-associated beta-galactosidase staining was performed in the 96well plate was performed as previously described [9, 16] and positive cells were individually counted per well.
